# Acknowledgment to Reviewers of *Vaccines* in 2021

**DOI:** 10.3390/vaccines10020213

**Published:** 2022-01-29

**Authors:** Vaccines Editorial Office

**Affiliations:** MDPI AG, St. Alban-Anlage 66, 4052 Basel, Switzerland

Rigorous peer-reviews are the basis of high-quality academic publishing. Thanks to the great efforts of our reviewers, *Vaccines* was able to maintain its standards for the high quality of its published papers. Thanks to the contribution of our reviewers, in 2021, the median time to first decision was 17 days and the median time to publication was 39 days. The editors would like to extend their gratitude and recognition to the following reviewers for their precious time and dedication, regardless of whether the papers they reviewed were finally published:
Abate, AndreaLand, Kenneth C.Abbate, Jessica L.Landolt, Gabriele A.Abraham, RachyLang, FengchaoAbravanel, FlorenceLange, AndrzejAbril, CarlosLangermans, Jan A.M.Acheson, AshleyLaranjo, MartaAcquasaliente, LauraLarrous, FlorenceAdadi, PariseLarsen, KristianAdamovicz, Jeffrey J.Lasrado, NinaadAdan, AnaLau, Ho Yin EricAdunyah, Samuel E.Lau, Joseph Tak-faiAggarwal, MeghaLavender, KerryAgha, SohailLavoie, Kim L.Agmon-Levin, NancyLaw, John Lok ManAgostini, SimoneLe Doaré, KirstyAgostinis, ChiaraLe Pendu, JacquesAguinaga-Ontoso, InésLe, Hoang-ThanhAgyei-Baffour, PeterLeavenworth, Jianmei W.Ahad, AbdulLeclerc, DenisAhlenstiel, ChantelleLecureux, JonathanAhmad, KhurshidLedgerwood, Emily DesmetAhmad, SajjadLee, ChoonghoAhmadian, GholamrezaLee, HowardAisa, RosaLee, Hye-raAjejas, JuliaLee, HyunjuAkbar, Sheikh Mohammad FazleLee, JihyeAkbulut, SamiLefebvre, FrancoisAlaimo, AlessandroLegaz, IsabelAlbendín-García, LuisLei, DylanAlberola, JuanLeitão, Jorge H.Albrecht, DavidLeng, AnliAlcolea, Pedro J.Leoncini, LorenzoAlderson, MarkLeón-Rubio, Jose MaríaAlexander, H. DenisLey, KlausAlfano, GaetanoLeyson, ChristinaAli, Hayssam M.L’homme, LaurentAlkan, MichaelLi, AngAllain, Jean-PierreLi, GuangyuAl-Lami, FarisLi, JieAllegaert, KarelLi, LiAllington, DanielLi, Mei-LingAl-Maharik, NawafLi, ShitaoAlmazan, ElbertLi, XinboAlsaggaf, RotanaLi, YonggangAltieri, MartaLian, Ie-BinAltmann, DannyLiang, BinhuaAmarilla, Alberto A.Liang, Xue-HaiAmarowicz, RyszardLiao, YifeiAmodio, EmanueleLichner, ZsuzsannaAndonov, AntonLichtenauer, MichaelAndréani, JulienLichtenwalner, AnneAndré-Fontaine, GenevieveLieber-Tenorio, ElisabethAndreoni, FrancescaLiebman, MichaelAndreotti, GiuseppinaLiechti, George W.Angelillo, ItaloLigat, GaetanAngelini, CorradoLim, Hui YinAngius, FabrizioLim, Siew PhengAnisimov, Andrey P.Lin, Chung-YingAnsa, BenjaminLin, Chyongchiou J.Anticoli, SimonaLin, HaishuAntoñanzas, FernandoLing, JiaxinAntonyuk, SvetlanaLingwood, DanielAntunes, JoanaLinial, MichalAnwar, FirozLio, DomenicoAranda, Carlos J.Lippke, SoniaAranday-Cortes, ElihuLiu, HaolinArciszewski, Marcin BartłomiejLiu, HejunAremu, OlatundeLiu, HuitaoAremu, TaiwoLiu, MargaretArena, FabioLiu, SaifeiAriani, AlaricoLiu, Shih-JenArmengot-Carceller, MiguelLiutsko, LiudmilaArnason, GardarLivaniou, EvangeliaArora, Arinder K.Lo Presti, ElenaArora, GunjanLo, Shih-YenArukha, Ananta PrasadLoconsole, DanielaAsakawa, MitsuhikoLogue, JamesAsare, MattLoh, JacelynAsdaq, Syed M.B.Lombardo, GiorgioAseervatham, JayaLomonossoff, George P.Ashbrook, AlisonLong, BradleyAsin, JavierLong, Carrie MaeAttia, SamehLong, Chiau MingAttri, KuldeepLong, MengxianAtzori, LauraLópez, DanielAudet, JonathanLópez-Siles, MireiaAxiotis, EvangelosLorgelly, PaulaAyala, JuanLoubert, LindaAziz, FaisalLozano-Paniagua, DavidAziz, KhaledLu, FengminAzuma, ChiekoLucas, SebastianAzzi, AlbertaLucas-Domínguez, RutBabiuk, LorneLuciani, MirellaBabiuk, ShawnLukov, GeorgiBachmann, MartinLundstrom, KennethBae, SeungJinLunel-Fabiani, FrançoiseBahmad, HishamLungu, Claudiu N.Bahrs, ChristinaLüth, StefanBăicuș, AndaLv, HuibinBaicus, CristianLynge, ElsebethBailey, Adam LeeMacaluso, GiusiBaindara, PiyushMaccagno, MassimoBaiu, Dana CarinaMacchi, FrancescaBaj, AndreinaMacGregor, CasimirBaker, DavidMachida, MasakiBakrania, BhavishaMaciver, SutherlandBala, SaikatMacRaild, ChristopherBalaraman, VelmuruganMadamsetty, Vijay SagarBalasubramaniam, MuthukumarMadsen-Bouterse, Sally A.Baldanzi, GianlucaMaeda, NoriakiBalieva, Flora N.Maekawa, ShunBall, Patrick A.Magariños Ferro, BeatrizBalla, Keir M.Maglie, RobertoBalzani, AgneseMagnavita, NicolaBamford, ConnorMagurano, FabioBanadyga, LoganMahmoud, AymanBanerjee, KasturiMaietti, ElisaBankala, KrishnarjunaMaisey, KevinBankwitz, DorotheaMajer, AnnaBannantine, John P.Malchiodi, Emilio L.Bansal, AnjuMaleki Vareki, SamanBao, YongmingMalinauskas, RomualdasBaquero, Monica M.Malleret, BenoitBara, JeffreyMallo, NataliaBarata, PedroMaloberti, AlessandroBarbezange, CyrilMamidi, MuraliBarchitta, MartinaManam, SrikanthBarrett, AlanMancianti, FrancescaBarroso, HelenaMandel, MichaBaršić, BrunoMandilara, GeorgiaBartholomeeusen, KoenManfredi, MarcelloBartok, EvaMangsbo, Sara M.Bartoszek, AgnieszkaManning, JessicaBärwolff, GünterManzoli, LambertoBashyal, SantoshMaraolo, Alberto EnricoBastos, ArmandaMarasca, ClaudioBasu, SankarMarascio, NadiaBasu, SaumikMarć, Małgorzata AnnaBatchelor, PaulMarchesi, AlessandraBates, John T.Marchesi, FedericoBatista-Duharte, AlexanderMarcilla, AntonioBatra, HimanshuMarco, DaddaBecker, Katrin AnneMarenzoni, Maria LuisaBehnam, Mira A.M.Marguello, Miguel SantibañezBélanger, KasandraMarimon, Jose MariaBeliakova-Bethell, NadejdaMarinelli, EnricoBeloukas, ApostolosMarinos, GeorgiosBelov, GeorgeMarion, Jason W.Beltowski, JerzyMarotta, ClaudiaBenati, DanielaMarsilio, FulvioBencúrová, ElenaMarson, PieroBen-David, BoazMartín, VerónicaBenevene, PaulaMartinelli, ChiaraBenfield, David A.Martin-Galiano, Antonio J.Benítez-King, GloriaMartín-García, ElenaBennardo, LuigiMartinho, José PedroBennett, JulieMartins, AndrewBenos, Alexis S.Martins, Jorge N.R.Bentel, Jacqueline M.Marzi, AndreaBera, SoumenMarzuillo, CarolinaBeran, JiriMascarenhas, MayaBercovier, HerveMasiulis, MariusBerezin, Eitan NaamanMaslow, Joel N.Bergh, ØivindMasoller, NarcísBerkowska, Magdalena A.Masresha, BalchaBerri, FatmaMastalerz-Migas, AgnieszkaBertau, MartinMastino, AntonioBerthelot, LaurelineMastrangelo, PeterBertram, MirandaMatarese, GiovanniBertzbach, Luca DaniloMateos-Hernández, LourdesBerzero, GiuliaMathieu, CyrilleBestetti, Reinaldo B.Mathioudakis, Alexander G.Bettencourt, PauloMatsuo, EikoBettridge, JudyMatsuo, KazuhikoBeyi, Ashenafi F.Mattei, FabrizioBezirtzoglou, EugeniaMattiuzzo, GiadaBhagavathula, Akshaya SrikanthMattner, JochenBhargava, AditiMattoscio, DomenicoBhattacharya, AnannyaMatveeva, OlgaBhattacharyya, SanchariMaurin, MaxBhawal, Ujjal KumarMaurya, ShailendraBhusetty Nagesh, Prashanth KumarMayner, LidiaBhutta, Mahmood FazalMazor, OhadBianchi, Francesco PaoloMazurov, Dmitriy V.Bianco, FrancescoMcAndrew, SiobhanBidmos, FadilMcBride, JohnBielefeldt-Ohmann, HelleMcElroy, Jane A.Bikov, AndrasMcIntosh, E. David G.Bins, Adriaan D.McKinstry, Karl KaiBiondo, CarmeloMcLaren, James E.Birlea, MariusMcLucas, BruceBiswas, SudiptaMcVey, DavidBlair, CarolMedina, Gisselle N.Blanco-Lobo, PilarMei, Kuo-ChingBlasco, Jose MariaMellinghoff, SibylleBleilevens, ChristianMénard, HervéBlunt, MatthewMendonça-Lima, LeilaBobkova, MarinaMereu, AlessandraBocedi, AlessioMerino, Jose JoaquinBock, Claus-ThomasMerritt, RowenaBock, WolfgangMertig, AngelaBogers, Willy M.J.M.Mesch, GustavoBogucki, JacekMesquita, JoãoBohannon, KevinMestres, ConxitaBöhmer, Merle M.Metovic, JasnaBohmwald, KarenMeurens, FrançoisBommireddy, RamireddyMeyer, SaschaBonay, MarcelMeyerhans, AndreasBongiovanni, MarcoMeysamie, AlipashaBonini, SergioMhatre, SiddhitaBoquet, DidierMiao, QiuhongBorges, MargaridaMichos, Athanasios G.Borkow, GadiMigliore, SergioBoroń-Kaczmarska, AnnaMigliorini, PaolaBos, NicoMilani, Gregorio P.Bose, Rajendran J.C.Milardi, DaniloBotgros, RaduMilavetz, BarryBöttcher, SindyMilella, MicheleBottero, JulieMiller, ElizabethBouman, JudithMingorance, JesúsBourdon, EmmanuelMiniero, Daniela ValeriaBowen, DeborahMinutolo, AntonellaBraack, MalteMirza, ImranBracco, EnricoMishra, AartiBranca, Jacopo Junio ValerioMishra, ManishBrandenburg, Lars-OveMishra, PriyankaBrandt, SabineMishra, SanketBrieger, AngelaMishto, MicheleBrochot, EtienneMitov, MihailBroecker, FelixMitra, Amal K.Brogi, SimoneMitsios, AlexandrosBrogna, BarbaraMiyagi, EtsukoBrooks, Benjamin D.Moccia, MarcelloBrown, Catherine M.Modenese, AlbertoBrown, Mark A.Modhiran, NaphakBrown, Richard J.P.Mohammed, AltafBrugliera, LuigiaMohsen, MonaBrunton, LucyMoiré, NathalieBruzzese, DarioMojumdar, KamalikaBruzzese, FrancescaMolarius, AnuBsisu, IsamMolenaar, Remco J.Buchta, ChristophMomotani, EiichiBuddaseth, SalmaMongin, DenisBugarin, AlejandroMontalti, MarcoBukrinsky, MichaelMontalto, LuigiBungau, SimonaMonteiro, SílviaBurckhardt, ChristophMontes, NuriaBurge, RusselMontgomery, Courtney GrayBurián, KatalinMontoya-Alonso, José AlbertoBurton, Zachary F.Moore, Justin XavierBuseyne, FlorenceMooyottu, ShankumarBuxton, SamuelMorales Suárez-Varela, María M.Byadgi, Omkar VijayMorales, MaríaCaballero, PabloMora-Montes, HectorCabaret, JacquesMorciano, GiampaoloCabeda, José ManuelMorea, DonatoCadeddu, ChiaraMoreira, Ana Maria Da PalmaCai, ShuoweiMoreno, EstherCain, Derek W.Moretti, SoniaCalagna, GloriaMori, MasaakiCalina, DanielaMorpurgo, MargheritaCallendret, BenoitMorrison, IvanCalligari, PaoloMortara, LorenzoCampbell, Nicole K.Mosconi, GiovanniCampello, ElenaMotamed, CyrusCampioni, PaoloMouchtouri, Varvara A.Campo, FlaminiaMould-Quevedo, Joaquin F.Campus, GuglielmoMousa, Jarrod J.Camus-Bouclainville, ChristelleMoutschen, MichelCantini, FrancescaMoya Higueras, JorgeCanuti, MartaMsallam, Rasha A.Caputo, AntonellaMu, QinghuiCara, AndreaMueller, Judith E.Carbajo-Lozoya, JavierMukai, HidehitoCardoso, FernandoMukai, TomoyukiCardoso, LuísMukherjee, AnupamCareem, FaizalMukherjee, JeanCarestia, MariachiaraMukherjee, SoumyadeepCarlo, SignorelliMukiibi, RobertCarmagnola, DanielaMukundan, SanthoshCarmignani, MarcoMulcahy, Grace M.Carneiro, LuizMulder, BobCarnell, GeorgeMuller, MandyCarr, Robert A.Müller, MartinCarrasco, AdrianoMüller-Trutwin, MichaelaCarreras, JoaquimMúñez, ElenaCarvalho, EdmundMuniraju, MuraliCascone, GiuseppeMuñoz-Barroso, IsabelCassimos, DimitriosMura, CameronCastaldi, SilvanaMurale, Dhiraj P.Castellazzi, MassimilianoMurata, ShiroCastiello, LucianoMurdaca, GiuseppeCastiglia, PaoloMurmura, FedericaCastro Esteves, Pedro JoséMurphy, DenisCattalini, MarcoMurugan, Natarajan ArulCedrone, FabrizioMurugan, RajagopalCerón-Carrasco, José P.Murugan, SengottuvelanCerqueira, LauraMusalgaonkar, SharmishthaChabot, Donald J.Mutinelli, FrancoChackerian, BryceMutz, PascalChaganty, BharatMuxel, Sandra MarciaChaguza, ChrispinMyc, AndrzejChakrabarti, JayatiMytych, JenniferChan, Kwok-HungNabavi, Seyed MassoodChan, MartinNacher, MathieuChandrashekar, AbishekNaguib, MahmoudChao, Yu-ChanNair, SmitaChappell, KeithNakamizo, TomokiChatterjee, SampurnaNakamura, KyoheiChatterjee, SatabdiNalos, MarekChavez-Calvillo, GabrielaNam, TaewooChawla-Sarkar, MamtaNapoli, ChristianCheloha, RossNardini, RobertoChen, ChuanNaruse, HiroyukiChen, Chung-MingNarzisi, AntonioChen, GangNasar, FarooqChen, Kuo-HuNath, NiharikaChen, LechuangNaylor, AndrewChen, ShanquanNazki, SalikChen, ShichengNde, Pius N.Chenais, BenoitNeely, Melody N.Cheng, ZishuoNeerukonda, Naga Venkata SabarinathChesneau, ChristopheNegri, DonatellaChiancone, FrancescoNekhai, SergeiChies, JoséNellimarla, SrinivasChiffi, DanieleNeter, EfratChina, BernardNetesov, SergeyChinnici, GaetanoNeuman, BenjaminChinta, Krishna C.Newman, John F.Chiostri, MarcoNg, Qin XiangChiozzini, ChiaraNghia, Truong PhuocChlichlia, KaterinaNghiem, SonCho, Do-YeonNgunjiri, JohnChoi, Chang WonNguyen, Duc-HiepChoi, Hyo GeunNí Cheallaigh, ClíonaChoi, Seong-ONicoletti, LoredanaChokshi, AalapNicoli, FrancescoChong, RogerNikbakht Nasrabadi, AlirezaChoudhary, AlokNikolaou, ElissavetChow, Franklin W.N.Nikolich, MikeljonChowdhury, Imran HussainNishizono, AkiraChowdhury, ShafiqulNissapatorn, VeeranootChristodoulides, MyronNistal-Villán, EstanislaoChua, Eng GuanNitta, TakayukiChuang, Hung-YiNogales, AitorChun, Kwang-HoonNoh, Jae MyoungChung, Hee-ChunNomizo, TakashiÇiftçi, Halil İbrahimNovick, DanielaCimmino, FloraNovikov, AlexanderCingolani, MarianoNovo-Veleiro, IgnacioCingoz, OyaNowak, GlenCipolloni, LuigiNtanasis-Stathopoulos, IoannisCircella, ElenaNunez, AlejandroClark, AndrewO’Connor, DanielClarke, PennyOdone, AnnaClemens, MarkOey, MelanieClement, CristinaOhkuri, TakayukiCliment, JoanOkamoto, ShigefumiCochis, AndreaOkazaki, KatsunoriColebunders, RobertOkumura, NobuakiColeman, ChristopherOliveira, GuilhermeColla, EmanuelaOliver, JoAnneCollins, CatherineOlivetta, EleonoraCollura, AdaOlsen, DanielColman, RickiOnorato, LorenzoComas-Garcia, AndreuOnyilagha, ChukwunonsoComas-Garcia, MauricioOpata, MichaelConn, David BruceOrd, KeithConners, GregoryO’Reilly, MaryConsole, LaraOrtega, Joseph ThomasConte, FerruccioOrtega, Miguel A.Conti, PioOrtega, NievesContu, PaoloOrusa, RiccardoConway, BrianOsuna, EduardoCoombs, KevinOtani, NaruhitoCoppeta, LucaOu, Chun-QuanCordero, OscarOyekale, AbayomiCortes-Cerisuelo, MiriamOzaki, ShozoCosby, Sara LouiseOzdarendeli, AykutCosseddu, Gian MarioPakpour, Amir Haji AghaCossu, DavidePal, DhimanCostanza, MassimoPalaiodimos, LeonidasCoudert, JeromePalaniswamy, SaranyaCriado-Alvarez, Juan JoséPalazón-Bru, AntonioCrooks, GeorgePalladino, RaffaeleCrupi, Mathieu J.F.Palmberger, DieterCruz Hervert, Luiz PabloPalmisano, AlidaCugliari, GiovanniPalumbo, MarcoCull, BenjaminPan, XiaolingCusick, Matthew F.Pandey, RajanCuypers, BartPanella, MassimilianoDa Fonseca, Flavio GuimaraesPang, IrisDa Silva, Henrique BorgesPapadopoulos, GeorgiosDahiya, SatinderPapandreou, NikolaosDal Col, JessicaPapazisis, GeorgiosD’Amelio, RaffaeleParan, NirDamiani, DanielaParente, PaolaDamiani, GiovanniParisini, EmilioDanovaro-Holliday, CarolinaPark, Eun JeongD’Apice, LucianaPark, Jung-EunDarrow, JonathanParronchi, PaolaDatta, Prasun K.Parvate, AmarDavidson, IritPascutti, Maria FernandaDavies, Anita A.Patching, Simon G.Dawen Yu, EstherPatel, SonalDawood, Mahmoud A.O.Patrianakos, AlexandrosDe Alwis, Ruklanthi (Rukie)Pavelko, Kevin D.De Andrés Calle, RocíoPavone, PieroDe Araújo, Tânia MariaPaweska, JanuszDe Bonis, SalvatorePeabody, DavidDe Bragança Pereira, Carlos AlbertoPechous, RogerDe Jonge, MarienPeghin, MaddalenaDe La Maza, Luis M.Pei, Yonggangde la Torre, Juan C.Peixe, PaulaDe Perio, MariePejchal, JaroslavDe Robertis, MariangelaPellegrinelli, LauraDe Sanctis, Juan BautistaPépin, MichelDe Silva, PushpamaliPerch, MichaelDe Simone, Salvatore GiovanniPerche, FedericoDe Vita, AlessandroPerdomo, JoseDe Waele, JorritPereira, MariaDeback, ClairePerez, FranciscoDebela, Ahmed M.Perez, Javier J.Debin, TianPérez-Bermejo, MarcelinoDeere, JessePerrella, AlessandroDegiannis, DimitriosPerricone, CarloDel Fresno Sánchez, CarlosPessela João, BenevidesDel Mistro, AnnarosaPetraityte-Burneikiene, RasaDel Riccio, MarcoPetrillo, MarcoDelbue, SerenaPetrini, StefanoDelcea, CameliaPezzoni, GiuliaDelhon, GustavoPfaller, ChristianDepken, CraigPflughoeft, KathrynDeplanque, DominiquePhanse, YashdeepDeStefano, Christin B.Phogat, SanjayDevasundaram, SanthiPiantino Ferreira, Antonio JoséDevaurs, DidierPiedra, Pedro A.Devy, ZismanPierannunzio, DanielaDewals, BenjaminPileggi, ClaudiaDhakal, SantoshPimpinelli, FulviaDhungyel, OmPinacchio, ClaudiaDi Bella, SantinaPincus, Matthew R.Di Bona, DaniloPincus, SethDi Domizio, JeremyPires, CarlaDi Gennaro, FrancescoPiscopo, MarinaDi Giovanni, PamelaPiskin, SenolDi Giuseppe, GabriellaPiu, SahaDi Guilmi, Anne-MariePlans-Rubió, PedroDi Maria, EmilioPlant, EwaDi Martino, GiuseppePokorski, MieczyslawDi Profio, FedericaPoluzzi, ElisabettaDi Stefano, MariantoniettaPoma, AdolfoDias, JoanaPomara, CristoforoDíaz-Rosales, PatríciaPomastowski, Paweł P.Dielschneider, RebeccaPoniedzialek, BarbaraDiemberger, IgorPoo, HaryoungDiesfeld, KatePoole, Brian D.Dijkstra, FrederikaPopa, Mircea IoanDimitrov, KirilPouliakis, AbrahamDing, LingwenPoveda, CristinaDixon, LindaPozzi, CeciliaDkhar, Hedwin KitdorlangPrakas, PetrasDocea, AncaPrasad, AbhishekDolan, Brian P.Pratesi, FedericoDolinski, DariuszPrice, PatriciaDomenech, AnaProkop, Jeremy W.Domingo, CristinaProspero, EmiliaDomnich, AlexanderProtopopescu, CameliaDonadu, MatthewPrüller, FlorianDondi, AriannaPsarra, KaterinaDong, ChunhongPuig-Barberà, JuanDonini, MarcelloPulido, OlgaDörr, MarcusPunetha, AnkitaDory, DanielPurvis, RachelDos Santos Dias, LucasPustylnikov, SergeyDosoky, NouraPyo, Hyun MiDowd, Kimberly A.Pyzik, MichalDoytchinova, IriniQu, BingqianDrillien, RobertQuiñones-Mateu, Miguel E.Duarte, RicardoRachiotis, GeorgeDubé, MathieuRadcliff, Fiona JaneDubovi, EdwardRadin, MassimoDucatez, MarietteRaeven, RenéDuCros, PhilippRafaniello, ConcettaDuddempudi, Phaneendra K.Raftery, MartinDudhipala, NarendarRagan, IzabelaDulebenets, MaximRahimi, SiavashDumache, RalucaRajaiya, JayaDumitrescu, OanaRajkumar Singh, KalraDunn, JohnRajnik, MichaelDuplaga, MariuszRakotondrafara, AurelieDuque, HernandoRamadori, GiulianoDyer, ArthurRamasamy, RanjanDzimianski, JohnRamatchandirin, BalamuruganEbensperger, GermánRamchandani, DivyaEbina, HirotakaRamírez-Paredes, Jose GustavoEccher, AlbinoRamírez-Toloza, Galia A.Eckels, Kenneth H.Ramos-Morcillo, Antonio JesúsEdith Rojas, AnayaRani Banik, GouriEdwards, Emily S.J.Ranzato, EliaEhrhardt, MatthiasRao, GuodongEhtisham-ul-Haque, SyedRao, Praveen P.N.Eibl, Martha M.Rapi, StefanoEick, ChristianRapone, BiagioEl Hadi, HamzaRatajczak, MagdalenaEldridge, SandyRather, IrfanElena, CriscuoloRauf, MohdElguezabal, NataliaRaundhal, MaheshElharrak, MehdiRava, MartaEljaafari, AssiaRavindra, P. VeerannaElkrief, ArielleRay, SamriddhaEmran, Talha BinRaze, DominiqueEndo, MakotoRazzuoli, ElisabettaEngelmann, IlkaReal-Fernández, FelicianaEscaffre, OlivierReche, PedroEscudero-Pérez, BeatrizReece, JeanetteEspinosa, EmmaRegla-Nava, Jose A.Essani, KarimRegmi, KrishnaEsser, MarkReid, ScottEsworthy, Robert StevenReis, AnaFaaberg, KayRemmerswaal, Ester B.M.Fabris, MartinaRenu, SankarFadda, MartaReyes-del Valle, JorgeFaita, FrancescoReynolds, Matthew R.Fallarini, SilviaRiccò, MatteoFalzone, LucaRichards, Stephen J.Faquim-Mauro, Eliana L.Richardson, JenniferFara, Gaetano MariaRichner, Justin M.Farina, AntonellaRigby, MichaelFarnos, OmarRinnerthaler, MarkFarronato, MarcoRiquelme, ErickFarsang, AttilaRissanen, IlonaFaure-Dupuy, SuzanneRiva, GiuseppeFedele, GiorgioRizzo, AlessandroFeldman, CharlesRoach, Jared C.Feng, HuapengRobinson, Sally R.Feng, NingguoRobles-Marhuenda, AngelFernandes, Elizabeth S.Roby, Justin A.Fernandes-Pedrosa, Matheus De FreitasRoccetti, MarcoFernández-Alarcón, ClaudiaRocha Faustino, Ana IsabelFernández-Prados, Juan SebastiánRodrigues, Andreia C.M.Fernández-Soto, PedroRodríguez Ortega, Manuel JoséFerorelli, DavideRodríguez, FernandoFerraioli, GiovannaRoh, ChanghyunFerrantelli, FlaviaRohaim, MohammedFerrara, PietroRojo, Maria AngelesFerree, KarenRoldão, AntónioFerreira, DavidRolla, GiovanniFerreira, FernandoRolla, SimonaFiems, DieterRomano, FerdinandoFiester, SteveRomano, NiclaFife-Schaw, ChrisRong, Chan KuanFilippini, FrancescoRoni, MonzurulFilippou, Panagiota S.Ronsard, LaranceFiore, MariaRoot-Bernstein, RobertFirczuk, MałgorzataRosenthal, KenFischer, CarloRossetti, Carlos A.Fletcher, Nicola F.Roubalova, KaterinaFlisiak, RobertRouleau, MatthieuFlora, GaganRouthu, Nanda KishoreFol, MarekRouzine, IgorFonseca, Gregory J.Rovin, Richard A.Formenti, PaoloRowland-Jones, SarahForsyth, Donelson R.Roy, IndraniFossum, CarolineRübsamen, NicoleFossum, EvenRuggiero, AlessandraFowke, KeithRuggiero, GiuseppinaFoyle, LeoRuiz, MauriciFraguas-Sánchez, Ana IsabelRukambile, Elpidius J.Fraleoni-Morgera, AlessandroRuntuwene, Lucky RonaldFraley, Gregory S.Ruotsalainen, JanneFranchini, MassimoRussell, AlistairFranciosini, MariaRusso, MomtchiloFranco, DavidRuysschaert, Jean MarieFranzoni, GiuliaRužauskas, ModestasFreer, GiuliaRybka, JakubFregeneda-Grandes, Juan-MiguelRyter, KendalFreiberg, Alexander N.Sabbatucci, MichelaFreick, MarkusSabeta, ClaudeFrigo, LúcioSabir, HemmenFronteira, InesSachithanandham, JaiprasathFrossard, Jean-PierreSagnelli, CaterinaFrutos, RogerSahu, Kamal KantFry, LindsaySahu, RajnishFthenakis, GeorgeSaid, Elias A.Fujisawa, TakaoSakač, NikolaFuller, A. OvetaSakellari, EvanthiaFuller, Frederick J.Saksena, NitinFurlini, GiulianoSaláková, MartinaFuruya, TetsuyaSalali, Gul DenizGacesa, RankoSalerno, LauraGaikwad, HanmantSalio, MariolinaGaíllat, JacquesSallam, MalikGajdács, MárióSalman, MootazGala, Rikhav P.Salpini, RominaGalanis, AlexisSalvato, MariaGałas, AleksanderSalzman, Mark B.Galindo-Villegas, JorgeSamir, ParimalGallegos-Arreola, Martha PatriciaSamy, AbdallahGallo, GaetanoSánchez Crespo, MarianoGaman, Mihnea-AlexandruSanchez, David JesseGamazo, CarlosSandonà, DoriannaGamil, AmrSándor, Attila D.Gan, LydiaSannella, AlessandraGan, Samuel Ken-EnSantacroce, LuigiGanju, AdityaSantarem, NunoGanta, RomanSantos, Adriana O.Garcia-Dorival, IsabelSantos, JeffersonGarcia-Moreno, FranciscaSardu, ClaudiaGarcía-Muñoz Rodrigo, FerminSarkar, AmritaGarfinkel, DavidSarker, SubirGarijo Toledo, María MagdalenaSarría Santamera, AntonioGarner, Bianca L.Sasaki, HirakuGärtner, BarbaraSato, HiroshiGarufi, CarloSatoshi Kashiwagi, SatoshiGatto, FrancescaSattentau, QuentinGaudreault, Natasha N.Sattler, David N.Gazerani, ParisaSaunders, JohnGe, BaoxueSauter-Louis, CarolaGea-Banacloche, Juan CarlosSaviano, AngelaGellert, George A.Savoia, ElenaGenovese, CristinaSawanyawisuth, KittisakGergely, LajosSaxena, PoojaGerhard, MarkusSayed, Ibrahim M.Gershwin, LaurelSayedahmed, EkramyGessler, FlorianScapigliati, GiuseppeGetti, Giulia T.M.Scarpa, FabioGhilardi Lago, João HenriqueSchaefer, AlexandraGhodake, GajananSchetters, Sjoerd T.T.Ghosh, MrinmoySchirhagl, RomanaGhosh, PrithwiSchirtzinger, Erin E.Giacomelli, AndreaSchlecht-Louf, GéraldineGiamberardino, Paolo DiSchmaljohn, AlanGiammanco, AnnaSchmidt, MartinGianfredi, VincenzaSchmitt, AnthonyGiangaspero, MassimoSchmoll, DieterGiannella, LucaSchoenhagen, PaulGiantsis, IoannisSchulte, BiancaGieryńska, MałgorzataSchwarz, AnkeGilbert, Amy TurmelleSchwarz, LukasGilbert, LeonaSchwarzinger, MichaëlGilmour, StuartSchweizer, MatthiasGimenez Llort, LydiaSchwermer, HeinzpeterGimple, Ryan C.Schwetz, ZollnerGirard, MarcScioscia, CrescenzioGiudici, PaoloScomparin, AnnaGiungato, PasqualeScorpio, DianaGiuseppe, ArcangeliScott, DanielGladue, DouglasScott, DavidGnat, SebastianScotti, MarcusGobbi, GiulianaSeals, Brenda F.Godfroid, JacquesSebastian, RobinGodman, BrianSebina, IsmailGogoi, HimanshuSeed, SheilaGoldman, AlejandraSeike, MasahiroGómez Cerezo, Jorge FranciscoSelim, Abdelfattah M.Gomez Del Moral, ManuelSelvaraj, ArokiyarajGomez, Carmen ElenaSelvaraj, PeriasamyGomez-Casado, EduardoSenna, GianenricoGómez-Sebastián, SilviaSeo, GiwanGómez-Urquiza, Jose L.Seo, Ho SeongGonçalves-Pereira, JoãoSeo, Sang HeuiGoncharuk, AnatoliySeow, JeffreyGong, LanSessa, FrancescoGongal, GyanendraSeth, AnushreeGonzalez, GabrielSethi, GautamGonzález, María EugeniaSethi, ManveenGonzález-García, AlbertoSevdalis, NickGonzález-Granado, Luis IgnacioSharma, PiyushGonzalez-Parra, GilbertoSharma, PrashantGonzalez-Rodriguez, AlexandreSharma, ShwetaGonzalez-Scarano, FranciscoSharonov, George VladimirovichGoodla, LavanyaShaukat, AftabGoodman, Alan G.Shaw, AndrewGopal, RadhaSheikh, Abu BakerGordon, StuartSheikh, ImlakGori, DavideShelley, MackGorini, FrancescaShelton, HollyGouandjika-Vasilache, IonelaShemirani, AmirGould, Ernest A.Shen, YangGraham, Melanie L.Shenoy, Anukul T.Granero-Molina, JoseSherwani, Mohammad AsifGregorio, Cesare DeShestopalov, Alexander M.Greim, HelmutSheu, JimGrilli, RobertoShimek, Jo AnnaGrinnemo, Karl HenrikShimodaira, ShigetakaGris-Oliver, AlbertShin, Crystal S.Grizzi, FabioShin, Hyoung-shikGroff, JosephSibley, DavidGroschup, MartinSiggins, Robert W.Grose, CharlesSignori, AlessioGrosse, Scott D.Sikalidis, AngelosGrossegesse, MaricaSiltz, Lauren A.Grossman, ZviSilva, AbigailGrubczak, KamilSilva, EdianeGrubman, MarvinSilva, Marcelo SousaGrubor-Bauk, BrankaSilva, Severino Jefferson RibeiroGrześk, GrzegorzSilva-Júnior, AbelardoGu, HaidongSilvestri, GiovanninoGudey, Shyam KumarSimecka, Jerry W.Guerriero, IlariaSimões, João Carlos CaetanoGuest, Johnathan D.Simões, Margarida PiresGuilherme, LuizaSinganalur, Nagendrakumar BalasubramanianGuillen-Grima, FranciscoSingh, Amit K.Guimarães, Jorge A.Singh, Brajesh KumarGul, SherazSingh, KamalendraGulla, KrishnaSingh, MahimaGuo, HuichenSingh, SandeepGuo, SijinSingh, SarbjitGutfraind, AlexanderSinigaglia, AlessandroGuthmiller, Jenna J.Sioofy-Khojine, Amir-BabakGutierrez-Guerrero, AlejandraSiristatidis, CharalamposGutiérrez-Martin, César B.Skinner, JackHaase, Elaine M.Skirrow, HelenHaeder, Simon F.Skwarczynski, MariuszHahn, AlexanderSlonchak, AndriiHai, RongŚmiałek, MarcinHall, RobynSmith, RichardHall, Roy A.Smith, Todd G.Haller, BernhardSmola, JiriHamilton-West, ChristopherSmulski, SebastianHammerl, Jens AndréSneddon, JenniferHampson, IanSochocka, MartaHan, DohyunSokoloski, KevinHan, DongWoonSolís García Del Pozo, JuliánHan, Jae YongSominskaya, IrinaHang, JunSong, ShuangHans, AymericSonnino, GiorgioHantz, SébactienSoreq, LilachHarada, MamoruSousa, AngelaHarkiss, GordonSpann, KirstenHarris, Audray K.Speijer, DaveHarrod, RobertSpellerberg, BarbaraHaslwanter, DeniseSpencer, Juliet V.Hassandarvish, PouyaSperone, EmilioHatmal, Ma’mon M.Spinu, MarinaHavrdova, EvaSpiro, David J.Hawkes, DavidSprenger, KaylaHearn, Michael J.Spurgeon, MeganHeegaard, Peter M. H.Sridhar, SiddharthHefferon, Kathleen L.Staats, Herman F.Hegde, BinduStanberry, Lawrence R.Hegele, Richard G.Stapleford, KennethHeidari, MohammadStasi, AlessandraHeller, E. DanStauber, RolandHelsloot, IraSt.Cyr, John A.Henderson, RorySteele, DuncanHeneberg, PetrStefanini, LuciaHepojoki, SatuStefanoff, PawelHerchenröder, OttmarSteffen, DavidHernaez, BrunoSteinberg, Julia R.Hernandez, JesusSteinborn, MichaelHernández-Breijo, BorjaSteiner, MargareteHernández-Castro, RigobertoSteinhagen, DieterHerod, MorganSteinke, DouglasHerrera, GuadalupeStephens, EdwardHerzberg, Philipp Y.Sternberg, RobertHess, Michael L.Stoecker, CharlesHeuft, Hans-GertStoiber, HeribertHigson, EdwardStone, MerlinHildt, EberhardStreck, André FelipeHinds, Lyn A.Strohfus, Pamela K.Hinkula, JormaSubbiah, JeevaHoang Vuong, QuanSue, Shih-CheHoelzle, Ludwig E.Sugawara, NorioHoffmann, BerndSugiura, KatsuakiHogenEsch, HarmSuliman, Hagir B.Holbrook, MichaelSullivan, John StephenHonko, AnnaSummerfield, ArturHooker, Angelo B.Sun, ChenyuHooper, Douglas C.Sun, XiangjieHorai, YoshiroSuperti, FabianaHorefti, ElinaSuryadevara, NaveenchandraHorii, ToshihiroSuschak, John J.Horn, MartinSwain, Jaya KumariHorobet, AlexandraSzabó, LászlóHorstman, KlasienSzymaniec-Mlicka, KarolinaHort, KrishnaSzymczak, AleksandraHorton, JohnTafforeau, LionelHostiuc, MihaelaTafuri, SilvioHour, Mann-JenTagliavia, MarcelloHozbor, DanielaTaitel, MichaelHsieh, Ming-KunTajima, MotoshiHuang, HuiTakahashi-Omoe, HiromiHuang, Jian-dongTakayama-Ito, MutsuyoHuang, JianshengTakeda, MakotoHuang, JunmingTalakić, EminaHuang, Sheng-WenTamayo, EstherHulme, JohnTana, ClaudioHung, Shuen-IuTandukar, SarmilaHurley, JamesTang, JianmingHussain, Ahmad FawziTanno, Luciana KaseHutchinson, KatrinaTarlinton, RachaelIampietro, MathieuTárraga Mínguez, RaúlIborra, SalvadorTārs, KasparsIborra-Egea, OriolTartey, SarangIde, KazukiTavares, JoanaIijima, NorifumiTaylor, Andrew W.Ikebuchi, RyoyoTazzari, MarcellaIlinykh, Philipp A.Tellier, MichaelIlling, Patricia T.Terán, EnriqueIlyinskii, Petr O.Thacker, NaveenImamura, RyuThannesberger, JakobImbimbo, BrunoTheron, JacquesImperatore, RobertaThomas, Roger E.Innes, CarrieThompson, Andrew J.Inoue, JunThompson, CraigIordanskiy, SergeyThompson, Dorothea K.Iorio, Ronald M.Tinker, JulietteIrigoyen-Camacho, María EstherTittarelli, AndreaIrina, ChelyshevaTiwari, PoojaIsaacs, StuartTodorov, SvetoslavIsel, CatherineTognetto, AlessiaIshigaki, YasuhitoToietta, GabrieleIslam, ArifulTomaszewska, EwaIsmael, Maria IsabelTonelli, MicheleIsola, GaetanoToniolo, Antonio Q.Isola, MiriamToranzo, Alicia E.Ita, KevinTorner, NuriaIturriza-Gomara, MirenToronto, Coleen E.Iwagami, MoritoshiTorres-Fernández, OrlandoIwata, KentaroTorres-Macho, JuanIwu, ChinweTorri, EmanueleIzumida, MaiToth, IstvanJain, SwatiToyos, Juan R. De LosJana, BimalTrafimow, DavidJankowski, MateuszTrama, FrancescoJanowski, MiroslawTrejbalova, KaterinaJansen Van Vuren, PetrusTrevisan, AndreaJanuszkiewicz-Lewandowska, DanutaTripathi, AshutoshJaramillo Ortiz, José ManuelTripathi, JitendraJavelle, EmilieTripathi, Jitendra KumarJeannot, EmilienTrollfors, BirgerJelinski, MurrayTrujillo, JessieJenkins, FrankTruman, Benedict I.Jenner, Dominic C.Trus, IvanJeong, Hye WonTsai, Ming-HorngJerkic, MirjanaTsergouli, KaterinaJerse, Ann E.Tsikouras, PanagiotisJesudhasan, PalmyTsioufis, KonstantinosJeulin, HélèneTung, Tao-HsinJiménez-Jiménez, Felix JavierTurbow, DavidJin, AndrewTurillazzi, EmanuelaJin, Jun-OTyagi, MuditJin, LiliangUcciferri, ClaudioJin, LingUddin, Md JamalJin, TengchuanUetrecht, CharlotteJohn, JacobUlbert, SebastianJohn, Sinu P.Ullah, Md AshikJohnson, Dylan M.Ulu, ArzuJohnson, Reed F.Urban, BrittaJones, ChrisUssowicz, MarekJones, IanUthaman, SajiJones, KennethUthman, OlalekanJones, MarjorieVabulas, R. MartinJones, MarkVaismoradi, MojtabaJones, PaulineValasoulis, GeorgeJones, Rodney P.Valicherla, Guru RaghavendraJose, MarcoValle, Manuel RodriguezJoshi, KamalVan Der Vossen, JosJoubert, AnnieVan Gisbergen, Marike W.Jung, KlausVan Gorp, E.C.M. (Eric)Jung, Tae SungVan Hemert, MartijnJungersen, GregersVan Keulen, Britt J.Junior, Joao P. AraújoVan Wagoner, Nicholas J.Jurisic, VladimirVaradarajan, JananiKabir, RussellVarani, LucaKaddour, HusseinVardasca, RicardoKaelber, JasonVarol, SerkanKagamu, HiroshiVarsani, ArvindKah, JanineVasile, FrancescaKaliamurthi, SatyavaniVasilyev, KirillKammerer, RobertVázquez-Domínguez, EvaristoKampen, Jarl K.Veiga, Ana Gorini DaKanatani, YasuhiroVelazquez-Salinas, LauroKanda, TatsuoVelin, DominiqueKandimalla, RameshVeltri, Giuseppe AlessandroKanekiyo, MasaruVenancio, Emerson JoséKang, Sang-MooVerros, George D.Kanginakudru, SriramanaVillalba-Montoro, RafaelKanoi, BernardVimercati, LuigiKaplan, ShaunaVirtaneva, KimmoKaraduta, OlegVistoli, GiulioKaranika, StylianiVisvizi, AnnaKarlsson Hedestam, Gunilla B.Visweswaran, Ganesh RamKarlsson, ErikVitale, ChiaraKarp, Jonathan D.Viviani, SimonettaKarpenko, LarisaVogiatzaki, ChrysanthiKarsten, Christina B.Voigt, Emily A.Kasprzak, RobertVon Horsten, Hans HenningKaszowska, MartaVrentas, Catherine E.Kato, FumihiroVuillefroy De Silly, RomainKato, HideoWada, KojiKatuchova, JanaWalduck, Anna K.Kaul, MarcusWang, Fun-InKaushik, RadheyWang, GuozhengKawaguchi, KazunoriWang, Hsin-YaoKawakami, MasanoriWang, Jiun-LinKawano, KouichiroWang, JunKazimírová, MariaWang, JunfengKazmi, ImranWang, KaiKeele, John W.Wang, MinghangKeeler, ShamusWang, Robert Yung-LiangKeller, FriederWannemuehler, Michael J.Kelly, Kevin M.Want, Muzamil YaqubKentaro, YamadaWard, Brian M.Kenzaka, TsuneakiWasniewski, MarineKersten, GideonWaterman, Paige E.Kesari, Kavindra KumarWaye, Mary M.Y.Kesselring, JürgWei, HongpingKeven, Joyal-DesmaraisWei, JinKhalid, HattafWei, RuifangKhan, KrishnenduWeina, Peter J.Khan, NaeemWeingartner, MagdalenaKhan, RabiaWeinrick, Brian C.Khanra, NandishWeiss, ChristelKhatami, AmenehWen, ConghuaKhatun, AminaWerner, EnriqueKhubchandani, JagdishWettstein, MartinKiara, HenryWhite, JacquieKido, HiroshiWieckowski, SébastienKikuti, MarianaWilliams, Edgar MarkKillian, Mary LeaWilliams, Jessica A.Kim, Gheun-HoWilliams, SusanKim, Hae WonWilliamson, Ethel DianeKim, Hei-sungWilm, MatthiasKim, HoonWilson, Alphus D.Kim, Hye KwonWilson, HeatherKim, In-JeongWilson, Jo LeanneKim, JongWilusz, JeffKim, Jung MinWissmath, BartholomäusKim, Ryong NamWohlleber, DirkKim, Sung-Kun (Sean)Wölfel, RomanKipar, AnjaWolfraim, LarryKirchenbaum, Greg A.Wolfrum, NinaKirui, JamesWong, Chi ChunKisa, AdnanWoods, Jon P.Kitagawa, SeiichiWoroniecka, KarolinaKlampatsa, AsteroWozniak-Knopp, GordanaKlann, Jeffrey G.Woźniakowski, GrzegorzKlaoudatos, DimitriosWu, HannahKlaumann, FranciniWu, Jack H.Z.Kleinsorge, ThomasWu, JianKluz, TomaszWu, JianxuanKo, Hae-JinWu, Mei X.Koekemoer, LizetteWubishet, BefikaduKohler, James J.Xia, ShuaiKokoszyński, DariuszXie, HangKolishetti, NageshXie, JianKomar, NicholasXu, Zhi PingKomase, KatsuhiroXue, GuangaiKommineni, NagavendraXue, MinKomori, AtsumasaYadav, Dharmendra K.Koncz, GáborYager, EricKontsevaya, IrinaYamamoto, KeiKöppen, KristinYang, MingKornilov, SergeyYaparla, AmulyaKorzeń, MarcinYe, YaozongKościelska-Kasprzak, KatarzynaYen, Cheng-FangKoseva, NeliYin, LiKoshizuka, TetsuoYoon, Kyungjae-andrewKosik, IvanYork, Ian A.Kosnik, MitjaYoshida, NoriakiKotozaki, YukaYoshio, SachiyoKouji, HaradaYost, Christian C.Kouokam, Joseph CalvinYoun, Bo-YoungKoutselas, IoannisYounes, HusamKovalchuk, AlexanderYoung, Sean G.Kovbasnjuk, OlgaYousefi, MortezaKowalska, MalgorzataYuan, LijuanKrishnasamy, GopinathYuan, MengKroeker, AndreaZahar, Jean RalphKrug, PeterZahm, Christopher D.Kuchar, Ernest PiotrZahn, Roland C.Kugelman, Jeffrey R.Zamperin, GianpieroKuhlmann, F. MatthewZapata, Juan CarlosKumagai, MakikoZappulla, David C.Kumar, AmitZavras, DimitrisKumar, HirdeshZefferino, RobertoKumar, ManishZeichner, Steven L.Kumar, RajZelger, BernhardKumar, SantoshZella, DavideKumar, VikashZeng, MinxiangKumararatne, DinkanthaZeynaloo, ElnazKumaraswamy Naidu, ChitralaZhang, DuoKumari, ShardaZhang, JianqiangKuryk, LukaszZhang, KunKusumoto, SojiroZhang, LizhouKwack, KyuBumZhang, ShuoKwakkel-van Erp, HannekeZhao, LinboKwan, JenniferZhou, DongmingLa Porta, RaffaeleZhou, FanLaabei, MaisemZhou, LangLabarère, JoséZidovec Lepej, SnjezanaLaboisse, Christian L.Zilliox, Michael J.Lada, Artem G.Zona, StefanoLaher, IsmailZost, SethLai, AlexanderZou, KeyuanLai, Michael M.C.Zumbrun, ElizabethLal, AmosZyśko, DorotaLambin, Philippe



